# Multiple Osteochondritis Dissecans in Multiple Joints

**DOI:** 10.1155/2021/8828687

**Published:** 2021-01-28

**Authors:** Takuto Takeda, Ryuichiro Akagi, Yusuke Sato, Takahiro Enomoto, Ryosuke Nakagawa, Seiji Kimura, Satoshi Yamaguchi, Satoru Nishikawa, Takahisa Sasho

**Affiliations:** ^1^Department of Orthopedic Surgery, Graduate School of Medicine, Chiba University, Japan; ^2^Department of Orthopaedic Surgery, Center for Advanced Joint Function and Reconstructive Spine Surgery, Graduate School of Medicine, Chiba University, Japan; ^3^Graduate School of Global and Transdisciplinary Studies, Chiba University, Japan; ^4^Nishikawa Orthopedics Clinic, Japan; ^5^Musculoskeletal Pain and Diseases, Center for Preventive Medical Sciences, Chiba University, Japan

## Abstract

**Background:**

Osteochondritis dissecans (OCD) rarely occurs in multiple joints. Furthermore, the existence of left-right asymmetric OCDs in different joints of the contralateral side of the body and lesions occurring with a temporal difference is rare. Here, we report a rare case with multiple OCDs sequentially detected in various joints. *Case Presentation*. The 15-year-old male patient was referred to our hospital for an OCD in the medial femoral condyle of the left knee. He had a history of an OCD in his right elbow, and his father had a history of surgically treated OCDs in both knees. One year and five months after, surgery was performed to the lesion in his left medial femoral condyle, a new OCD lesion occurred in the femoral trochlea of the same knee, which was again treated surgically. Five months after the second surgery, the patient returned with pain in the right knee, and an OCD on the right femoral trochlea was detected by an MRI scan. This lesion remained stable without any further restriction in physical activities for 17 months until detachment occurred and was again treated surgically.

**Conclusion:**

In cases with history and a family history of multiple OCDs, in particular, with a short stature, an MRI scan should be performed for the symptomatic joint to detect and treat the lesion before progression.

## 1. Background

Osteochondritis dissecans (OCD) is a disorder that affects the subchondral bone and potentially leads to detachment of cartilage and bone fragment with progression [1]. It is known to occur more frequently in male than in female. However, the exact etiology of the disease is unknown. Several theories have been described, including trauma, genetics, inflammation, nutritional imbalance, and vascular abnormalities [2–4].

Some cases with bilateral knee OCDs have been reported [5, 6], but there are no previous cases reported for more than three OCDs occurring in multiple joints. We present a case of a patient who presented with clinically symptomatic multiple OCDs which occurred in multiple joints, including the elbow and knee joint.

## 2. Case Presentation

**Figure 1:**
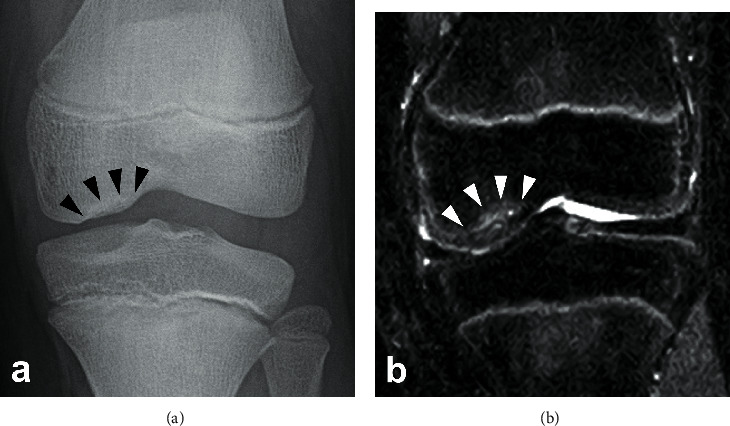
(a) Radiograph of the left knee showing Brückl's stage III OCD lesion in the MFC. (b) Short T1 inversion recovery (STIR) sequence MRI coronal image of the left knee indicating Nelson's stage II lesion in the MFC.

**Figure 2:**
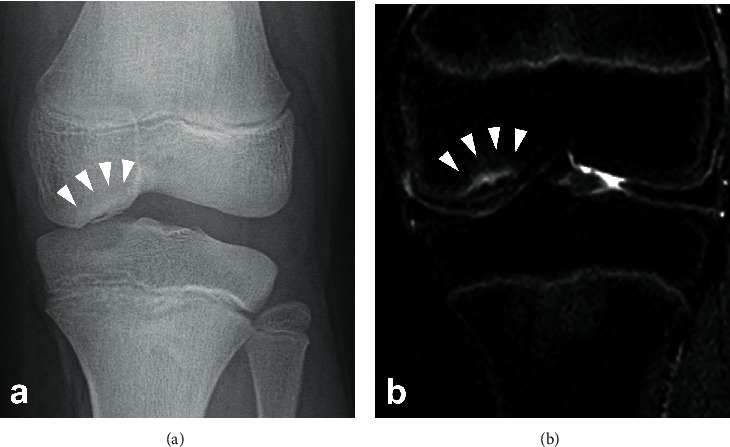
(a) Radiograph of the left knee showing Brückl's stage IV OCD lesion in the MFC. (b) STIR MRI coronal image of the left knee indicating Nelson's stage III lesion in the MFC.

A 15-year-old boy was referred to our department after the failure of a conservative treatment to an OCD lesion in his left knee. He presented at his orthopedic practitioner three months before referral to our department, complaining of pain in his left knee during sports activity such as baseball. At the initial visit, his height was 150 cm, which was below the -2 standard deviation (SD) cutoff (approximately -2.8 SD) of Japanese average of the same age, with a body mass index of 22. He had a history of OCD in the right elbow at the age of 11. The patient's father also had short stature (153 cm, -3.1SD) and had a history of OCDs in both knees, which was surgically treated after maturity. There was no other family history of OCD. There was no swelling or tenderness of the knee joint, and no abnormalities in the range of motion or joint laxity and stability were detected on physical examination. The boy did not have any pain during normal daily activities, including PE. An OCD of the medial femoral condyle (MFC) was detected by radiograph and classified as stage III, according to Brückl [7] (Figure 1(a)). The lesion was confirmed by magnetic resonance imaging (MRI) of the left knee, which was classified as stage II, according to Nelson's classification [8] (Figure 1(b)). He was treated conservatively by avoiding intense physical activity, while physical education (PE) at school was permitted. Despite the advertent treatment for three months, the radiographic finding of the lesion progressed to stage IV according to Brückl's classification (Figure 2(a)) and stage III according to Nelson's classification on MRI (Figure 2(b)). At this moment, he was referred to our department for surgical treatment.

**Figure 3:**
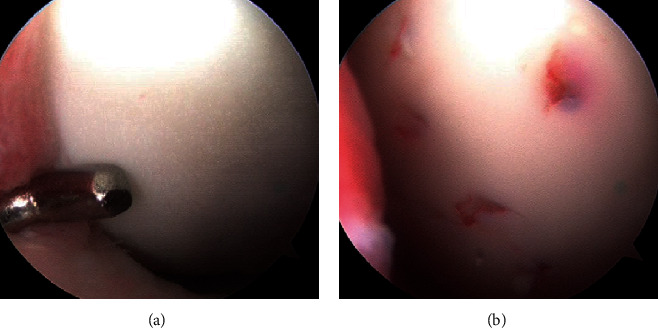
Arthroscopic finding of the left medial femoral condyle. (a) The lesion was identified by probing the cartilage surface and cleavage in the rim was detected. (b) Percutaneous drilling was performed in the lesion.

Since the patient was asymptomatic at the time he was referred to our department, he was treated conservatively for another three months but did not show any improvement in radiographic finding. Thus, we decided to perform surgical treatment. The operative treatment consisted of an initial arthroscopy. The lesion was identified since there was cleavage in the rim of the lesion by probing (Figure 3(a)), and the cartilage was slightly levitated, but not completely detached from the subchondral bone. The size of the lesion was 10 mm in width and 24 mm in length. Arthroscopic drilling with a 1.6 mm Kirschner wire from the surface of the lesion in MFC was performed, creating ten penetration deep enough to reach the subchondral bone underlying the lesion, for the purpose of inducing bleeding from the bone marrow to stimulate healing (Figure 3(b)). The lesion was confirmed to have achieved bony union by radiograph three months after surgery; sports activities were permitted.

**Figure 4:**
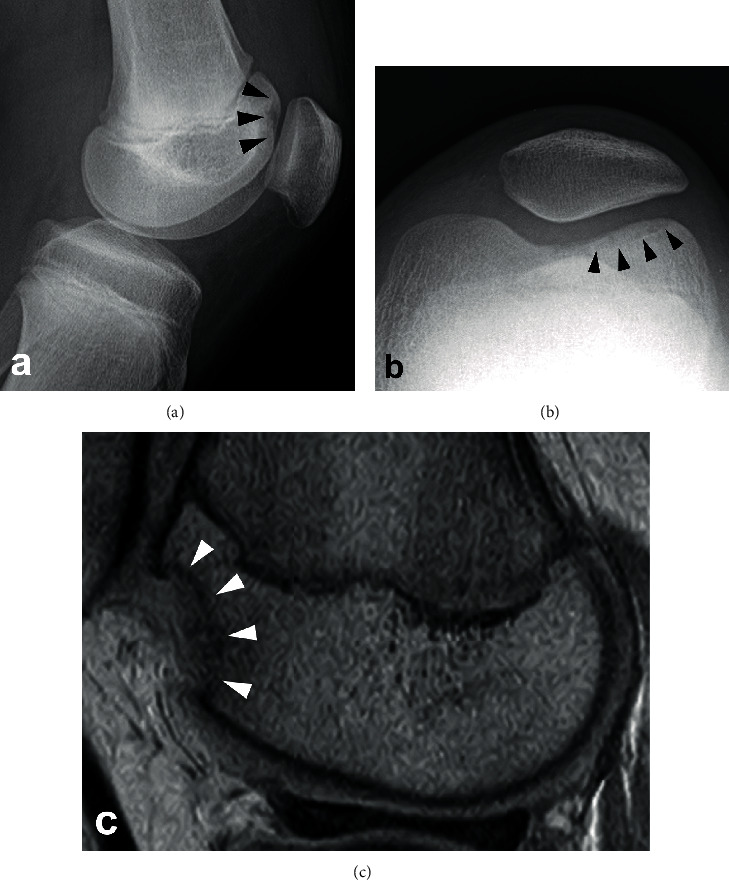
(a) Lateral view and (b) skyline view radiograph of the left knee showing Brückl's stage V OCD lesion in the lateral facet of the femoral trochlea. (c) Proton density-weighted MRI sagittal image of the left knee indicating Nelson's stage III lesion in the lateral facet of the femoral trochlea.

The postoperative course was successful until one year and four months after surgery when he fell and hit the left anterior knee. The radiograph taken immediately after this injury showed no abnormal findings. Since the patient started to feel pain on running, and since an event of locking of the knee joint occurred, we performed another radiographic examination one month after the injury but were unable to detect any abnormal findings, and the patient was conservatively treated. Four months after the injury, the knee became more frequently locked, and knee pain on flexion and swelling of the knee joint became evident, and another radiographic examination was performed. A defect in the lateral facet of the femoral trochlea that had been normal on the previous MRI before this event was detected by radiography, and the lesion was confirmed by MRI scan. The lesion was diagnosed as OCD in the femoral trochlea (Brückl classification stage V in the radiograph, and Nelson classification stage IV in MRI), and no kissing lesion was confirmed on the patella (Figure 4). The osteochondral fragment was treated surgically by fixation to the bed by four biodegradable pins. The fragment was confirmed to be stable by computed tomography and MRI scans, and the patient was allowed to return to sports at four months after surgery.

**Figure 5:**
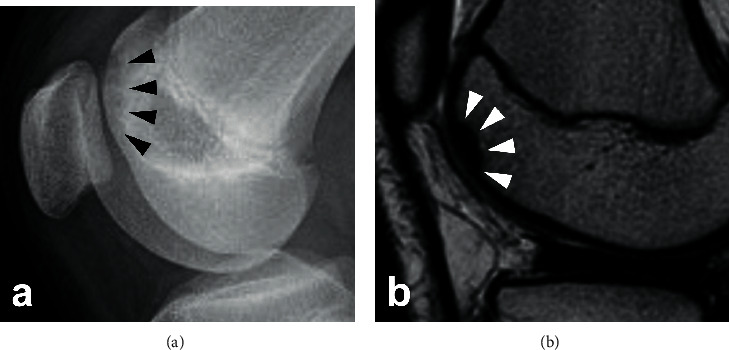
(a) Lateral view radiograph of the right knee showing Brückl's stage II OCD lesion in the lateral facet of the femoral trochlea. (b) T2-weighted MRI sagittal image of the right knee indicating Nelson's stage I lesion in the lateral facet of the femoral trochlea.

One month after returning to sports (5 months after fragment fixation surgery), the patient returned to the office complaining of pain in his contralateral knee during sports activities without any obvious history of injury. Radiography and MRI scan were performed for his right knee, and an OCD lesion of Brückl's classification stage II and Nelson's classification stage I OCD in the lateral facet of the trochlea was identified (Figure 5). The lesion was carefully observed and remained stable without any further restriction in physical activities. However, the lesion became unstable 17 months after its initial detection, and we performed an open reduction and internal fixation surgery under spinal anesthesia. The fragment was confirmed to have achieved union by three months after surgery.

## 3. Discussion and Conclusion

In the presented case, we experienced multiple OCDs occurring in various joints (4 lesions in the three joints), with the temporal difference in occurrence. We failed to detect one of the lesions in the early phase of the disease before detachment from the base and were required to perform surgery to treat the lesion.

In terms of the epidemiology of OCDs, bilateral lesions have been reported to be present in 7.3-29% [9–11] of OCDs of the knee. Bilateral knee OCDs might not be so rare, but a case of multiple OCDs occurring in multiple joints is a relatively rare condition. There were 21 cases of OCDs occurring in multiple joints previously reported in the literature, including bilateral knee cases [3, 5, 6, 12–25]. Among them, there was only one case that was reported for the existence of left-right asymmetric OCDs in different joints of the contralateral side of the body. We were able to identify some case reports regarding multiple OCDs that developed in a single knee joint. There were 10 cases in total. However, there was only one case which had two OCDs in the MFC and the patella-femoral compartment. It is also known that the main lesion of OCD is the classical site in the MFC, which accounts for 69% of the whole OCDs, and OCDs occurring in the trochlea is relatively rare [26].

Considering the timing of diagnosis of multiple OCDs according to the literature, there were 26 cases of multiple OCDs that mentioned the time point of diagnosis of each lesion. In most cases, multiple lesions were identified at the same time, and there were only 6 cases, which was diagnosed one after the other. It was reported that, in 40% of bilateral OCD cases, the incidentally diagnosed contralateral lesion was asymptomatic [9]. In other words, most lesions seem to occur around the same time regardless of symptoms, and a case that multiple symptomatic OCDs were occurring with a temporal difference is rare. In our case, all lesions were symptomatic at the time of diagnosis but were not necessarily detectable by radiographs until the progression of the disease. The presented case was rare in that (1) multiple OCD lesions in different joints (elbow and knees) were involved, (2) multiple lesions occurred with temporal difference instead of occurring at the same time, and (3) the femoral trochlea was affected bilaterally.

Several theories have been suggested for the etiology of OCDs, including ischemia of subchondral bone, trauma, repetitive microtrauma, genetic factor, metabolic disease, and inflammatory disease [2–4]. However, the cause of this disease is still under debate. In the case presented in this report, the patient and his father both had the phenotypic form of a short stature, with multiple OCDs. There are several case reports of multiple OCDs occurring in twins [3], within the family [12–14, 27], and some reported multiple OCDs occurring in a family with short stature [12, 27–29]. Genetic factors may play a major role in these cases.

There are a couple of limitations in the present paper. First, the lesion at the trochlea of the left knee might not be the OCD but traumatic osteochondral lesion. There were no abnormal findings on the patella on MRI, and lack of kissing lesion in the patella might support our diagnosis as an OCD. Second, an MRI exam of the right knee was performed only once before we detected the OCD in the right trochlea. Thus, we could not tell when the lesion developed.

In conclusion, in cases with history of OCD and a family history of multiple OCDs, in particular, with a short stature, an MRI scan should be performed for the symptomatic joint [30, 31] to detect and conservatively treat the lesion before progression.

## Abbreviation

OCD: Osteochondritis dissecans.
